# Scaling up oligogenic diseases research with OLIDA: the Oligogenic Diseases Database

**DOI:** 10.1093/database/baac023

**Published:** 2022-04-12

**Authors:** Charlotte Nachtegael, Barbara Gravel, Arnau Dillen, Guillaume Smits, Ann Nowé, Sofia Papadimitriou, Tom Lenaerts

**Affiliations:** Interuniversity Institute of Bioinformatics in Brussels, Université Libre de Bruxelles-Vrije Universiteit Brussel, Boulevard du Triomphe, CP 263, Brussels 1050, Belgium; Machine Learning Group, Université Libre de Bruxelles, Boulevard du Triomphe, CP 212, Brussels 1050, Belgium; Interuniversity Institute of Bioinformatics in Brussels, Université Libre de Bruxelles-Vrije Universiteit Brussel, Boulevard du Triomphe, CP 263, Brussels 1050, Belgium; Machine Learning Group, Université Libre de Bruxelles, Boulevard du Triomphe, CP 212, Brussels 1050, Belgium; Artificial Intelligence Laboratory, Vrije Universiteit Brussel, Pleinlaan 2, Brussels 1050, Belgium; Artificial Intelligence Laboratory, Vrije Universiteit Brussel, Pleinlaan 2, Brussels 1050, Belgium; Human Physiology and Sports Physiotherapy research group, Vrije Universiteit Brussel, Pleinlaan 2, Brussels 1050, Belgium; Interuniversity Institute of Bioinformatics in Brussels, Université Libre de Bruxelles-Vrije Universiteit Brussel, Boulevard du Triomphe, CP 263, Brussels 1050, Belgium; Hôpital Universitaire des Enfants Reine Fabiola, Université Libre de Bruxelles, Avenue Jean Joseph Crocq 15, Brussels 1020, Belgium; Center of Human Genetics, Hôpital Erasme, Université Libre de Bruxelles, Route de Lennik 808, Brussels 1070, Belgium; Interuniversity Institute of Bioinformatics in Brussels, Université Libre de Bruxelles-Vrije Universiteit Brussel, Boulevard du Triomphe, CP 263, Brussels 1050, Belgium; Artificial Intelligence Laboratory, Vrije Universiteit Brussel, Pleinlaan 2, Brussels 1050, Belgium; Interuniversity Institute of Bioinformatics in Brussels, Université Libre de Bruxelles-Vrije Universiteit Brussel, Boulevard du Triomphe, CP 263, Brussels 1050, Belgium; Machine Learning Group, Université Libre de Bruxelles, Boulevard du Triomphe, CP 212, Brussels 1050, Belgium; Artificial Intelligence Laboratory, Vrije Universiteit Brussel, Pleinlaan 2, Brussels 1050, Belgium; Interuniversity Institute of Bioinformatics in Brussels, Université Libre de Bruxelles-Vrije Universiteit Brussel, Boulevard du Triomphe, CP 263, Brussels 1050, Belgium; Machine Learning Group, Université Libre de Bruxelles, Boulevard du Triomphe, CP 212, Brussels 1050, Belgium; Artificial Intelligence Laboratory, Vrije Universiteit Brussel, Pleinlaan 2, Brussels 1050, Belgium

## Abstract

Improving the understanding of the oligogenic nature of diseases requires access to high-quality, well-curated Findable, Accessible, Interoperable, Reusable (FAIR) data. Although first steps were taken with the development of the Digenic Diseases Database, leading to novel computational advancements to assist the field, these were also linked with a number of limitations, for instance, the *ad hoc* curation protocol and the inclusion of only digenic cases. The OLIgogenic diseases DAtabase (OLIDA) presents a novel, transparent and rigorous curation protocol, introducing a confidence scoring mechanism for the published oligogenic literature. The application of this protocol on the oligogenic literature generated a new repository containing 916 oligogenic variant combinations linked to 159 distinct diseases. Information extracted from the scientific literature is supplemented with current knowledge support obtained from public databases. Each entry is an oligogenic combination linked to a disease, labelled with a confidence score based on the level of genetic and functional evidence that supports its involvement in this disease. These scores allow users to assess the relevance and proof of pathogenicity of each oligogenic combination in the database, constituting markers for reporting improvements on disease-causing oligogenic variant combinations. OLIDA follows the FAIR principles, providing detailed documentation, easy data access through its application programming interface and website, use of unique identifiers and links to existing ontologies.

**Database URL:**

https://olida.ibsquare.be

## Introduction

Understanding the genetic mechanisms underlying human diseases remains a key challenge in the field of human genetics. Studies investigating the association of human genetic variation with disease have brought forward an ever-increasing amount of evidence showing that the ‘one gene-one disease phenotype’ paradigm is often an oversimplification and is not adequate to explain the phenotype of individuals (1–3). It has been actually demonstrated that gene interactions, or epistatic effects, can influence the expression of our traits and also lead to disease or modulate its severity (4–6), with known examples including Bardet–Biedl syndrome (7), cystic fibrosis (8), Hirschsprung disease (9) and hereditary non-syndromic hearing loss (10). The discoveries of such epistatic effects and of the pathogenic role of the aggregation of multiple rare and common variants, as shown in neurodevelopmental disorders (11, 12), has led to the notion of a conceptual continuum in genetic diseases starting from rare monogenic disorders to oligogenic and more complex polygenic common diseases, which can be influenced further by environmental factors (13).

The detection of epistatic effects in human genetic disorders is linked with specific challenges and limitations, including the use of high-dimensional data sets requiring high computational resources and the challenging biological interpretation of the obtained results based on our current knowledge (14). Nevertheless, an increasing amount of data is being accumulated from pedigree and cohort analyses and functional studies on humans and animal models, as well as computational methods, all aiming to understand the synergistic mechanisms between genes leading to disease (3, 4, 14). Their results have indicated that genes contributing to the development of a disease tend to be closely biologically related, for instance, being linked with protein–protein interactions (PPIs), or involved in similar pathways and cellular processes linked to the disturbed phenotype (15–17).

The Digenic Diseases Database (DIDA) (18), created in 2015, served as an important first step to the realm of oligogenic diseases by collecting curated scientific information on digenic variant combinations, i.e. combinations of variants in two genes, leading to digenic diseases. Inspired by Schäffer (3), the DIDA authors made a first attempt to define inclusion criteria to select relevant DIDA cases: first, including only studies conducted in human patients, and, second, including digenic combinations linked with either experimental evidence of their pathogenic impact (functional evidence), evidence of the biological or clinical relationship between the genes (gene relationship evidence)—e.g. PPI, common pathways or involvement in the same disease—or genetic evidence of the variant combination co-occurrence for the observed phenotype based on a pedigree study (familial evidence). These criteria led to the inclusion of 258 combinations linked to 52 different digenic diseases, with Bardet–Biedl syndrome, familial haemophagocytic lymphohistiocytosis and familial long QT syndrome being the most represented disorders in the database. Two typical classes of digenic models were observed in DIDA (3, 4): the True Digenic cases, where variants in both genes are required to show symptoms of the disease, and the Composite, also called Monogenic plus modifier cases, where one variant is present in the primary most pathological gene and can alone convey symptoms of the disease, whereas the variant in the second less detrimental gene, i.e. the modifier gene, either affects the severity of the symptoms or the age of onset.

Since its launch, DIDA has been consulted frequently and successfully used as a benchmark dataset for a range of different machine learning methods aiming to predict and understand the cause of digenic diseases: VarCoPP (19), which predicts the pathogenicity of digenic variant combinations, and the Digenic Effect predictor that first differentiated between True Digenic and Monogenic plus modifier cases (20) and afterwards was expanded to a new method that differentiates between the original two classes and Dual Molecular diagnosis cases (21). Previously mentioned methods were incorporated in an online platform, ORVAL (22), along with annotations from public databases, to explore oligogenic variant combinations and predicted pathogenic gene networks in an individual. These tools have in turn been used successfully in scientific studies to analyse novel potential oligogenic cases (23–26), highlighting the importance of DIDA and marking the era of a new age of predictive tools tailored for more complex genetic diseases. At the same time, these advances demonstrated the need for a continuous and careful collection of new oligogenic data, which could start now expanding further in the genetic disease continuum than digenic diseases. Notwithstanding the usefulness of DIDA, its database and website structure was limited to digenic cases, and a thorough change in its architecture was needed to accommodate information on not only digenic but also other oligogenic (e.g. triplets) cases or combinations between copy number variations (CNVs) and other variants as described in the scientific literature (27). More importantly, the original criteria for the inclusion of oligogenic combinations in the database needed a serious re-evaluation, following the emergence of guidelines in reporting causative variants for genetic diseases (28), with a need of objective evaluation metrics reflecting the quality and strength of different types of evidence, genetic and functional, supporting their causality. The current work aims to resolve all these issues and more.

We present the OLIgogenic diseases DAtabase (OLIDA; https://olida.ibsquare.be/), which reinvents DIDA, containing newly curated and fully re-curated data, providing freely accessible information on all oligogenic variant combinations, i.e. variant combinations in multiple genes involved in an oligogenic disease, published in the scientific literature, including the digenic cases present in DIDA. Apart from single-nucleotide variations and small insertions/deletions (indels), OLIDA now also incorporates CNVs. The premise of OLIDA is to provide a reference repository of all available information on oligogenic variant combinations, with a rigorous quantification of the quality of each entry based on the empirical evidence that is provided to support it. For this purpose, an improved, transparent, thorough and more structured curation protocol specific for oligogenic variant combinations is introduced (see Materials and Methods), which assigns a confidence score to each oligogenic combination that depicts the strength of evidence supporting its link to a genetic disease. One of the most important advancements compared to DIDA is that special care is taken to consider the evidence of the joint pathogenic effect of the involved variants and genes in an oligogenic combination for an observed phenotype and, thus, opening the road to a discussion on how evidence on oligogenic diseases should be assessed and evaluated. OLIDA is built following the Findable, Accessible, Interoperable, Reusable (FAIR) principles on data management (29), with its unique identifiers per oligogenic combination, thorough documentation, commonly used domain vocabularies, qualified references to identifiers and meta-data, downloadable information and search through an application programming interface (API), clear data usage licence and its transparency in assigning confidence scores to the oligogenic combinations. OLIDA will lift the research of oligogenic diseases to a new level by providing an important resource for the future of precision medicine.

## Materials and Methods

### General premise of the confidence score for an oligogenic combination

In order to define the confidence score for an oligogenic variant combination, inspiration was drawn from Schäffer (3) and McArthur (28). The most important premise for accepting an oligogenic variant combination as causative for a particular disease phenotype is the presence of adequate evidence of the joint effect of the variants involved in the combination. More specifically, at first, adequate genetic evidence is needed showing that the segregation of these variants is linked to the disease phenotype and that the variants do not occur by chance. The genetic evidence can be obtained through the study of the segregation of the variants in a family (familial evidence) and/or the study of the frequency and effect of the variants in a healthy, unrelated population (statistical evidence). Second, adequate functional evidence is needed, both at the gene combination (gene evidence) and the variant combination levels (variant evidence), showing that the variants jointly have an impact on the normal function of the cell producing the observed phenotype and also that this impact is stronger or different than the impact of the individual variants. Oligogenic combinations defined as causal for an observed phenotype require both strong genetic and functional evidence.

For each type of evidence, we assign a strength or confidence level (expressed as the subsequent score value shown in parenthesis), generally defined as:

- Strong (3), if there is strong evidence and proof of the synergistic or additive effect of the oligogenic variant combination on the observed phenotype
- Moderate (2), if there is evidence of an effect of the oligogenic variant combination on the observed phenotype, but the described information or mechanism is not clear enough to provide a definite proof of oligogenicity
- Weak (1), if there is evidence of the relevance of the variant combination for the observed phenotype, but it is not certain that the involved variants are the only culprits for the studied phenotype or that the cause is indeed oligogenic
- Absent (0), if the information or evidence provided does not fulfil our criteria to assign at least a Weak score for a variant combination

The same score value has the same strength among all types of evidence (i.e. familial evidence, statistical evidence, gene functional evidence and variant functional evidence) and all types of scores. A higher score is always linked with stronger confidence in the causality of the variant combination for the observed phenotype. See Supplementary Data, File 1, for a detailed explanation of how each confidence score is defined per type of evidence.

### Manual curation procedure and manual curation scores

**Figure 1.**
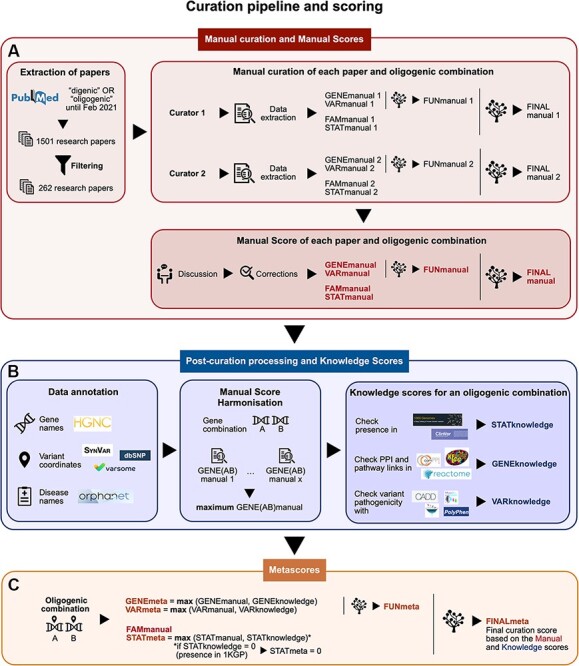
Summary of the curation pipeline for the creation of the (A) Manual scores, (B) Knowledge scores and (C) Metascores for each oligogenic combination. (A) Research articles were selected using the keywords ‘digenic OR oligogenic’ in PubMed (https://pubmed.ncbi.nlm.nih.gov/), leading from a total of 1501 articles to 262 articles after filtering (see Materials and Methods, Supplementary Data, File 1). Two different curators independently extracted information and curated each article, assigning Manual scores. These scores include the FAMmanual, STATmanual, GENEmanual and VARmanual scores. The last two are used in a decision tree to assign the FUNmanual score, while all of the scores are used in another decision tree to create the FINALmanual score (see Materials and Methods, Supplementary Data, File 1). A discussion then took place to reach a consensus for the Manual Scores. All oligogenic variant combinations were evaluated as separate entities with their own evidence, regardless of whether they were described in the same or different articles. (B) The data were then processed to formalize the available information (see Materials and Methods, Supplementary Data, File 1). The disease names were formalized using the Orphanet database (https://www.orpha.net/). The gene names were formalized according to the gene nomenclature guidelines from the HGNC database (33). The variants were processed by the software Synvar (http://goldorak.hesge.ch/synvar/), and the databases Varsome (34) and dbSNP (35), to obtain genomic coordinates. To correct for the literature bias, the GENEmanual score for each gene pair was harmonized among all articles by assigning the maximum GENEmanual found for that gene pair, and all affected scores (FUNmanual and FINALmanual) were recalculated. To compensate for missing information in the articles due to no prior access to current knowledge, Knowledge scores were assigned per oligogenic combination: the STATknowledge score by checking the presence of the oligogenic combination in the 1000 Genomes project (30) and ClinVar (36), the GENEknowledge score by checking the PPI and KEGG (38) or Reactome (39) pathway links of the involved genes and the VARknowledge score by using variant pathogenicity information from different pathogenicity predictors: SIFT (40), MutationTaster2 (41), CADD (42) and Polyphen2 (43). (C) Finally, both Manual and Knowledge scores are combined in order to create the confidence Metascores for each type of evidence STATmeta, GENEmeta and VARmeta scores—by assigning the maximum score found between their corresponding Manual and Knowledge score (see Materials and Methods, Supplementary Data, File 1). One exception occurs in this rule: if the STATknowledge is 0 due to the fact that the combination is found in an individual of the 1000 Genomes project, then it replaces the STATmanual and, therefore, the STATmeta is also 0. The same procedure as in the manual curation is then followed when decision trees were used to define the FUNmeta and FINALmeta scores.

Research articles were selected using the keywords ‘digenic OR oligogenic’ in PubMed (https://pubmed.ncbi.nlm.nih.gov/), discarding the studies: (i) not involving humans, (ii) not providing information about the exact variants involved, (iii) conducting statistics only at the gene level and (iv) containing chromosomal rearrangements and other large CNVs that span among many genes (Figure 1A). From a total of 1501 papers that were initially found in PubMed (in February 2021), 262 papers passed these criteria.

Two different curators were appointed per research article (Figure 1A) and independently extracted the relevant information (see Supplementary Data, File 1) and assigned curation scores to the combinations. A discussion then took place between them to reach a consensus in cases when differences arose either on the extracted information or the curation scores.

All oligogenic variant combinations were evaluated as separate entities with their own evidence, regardless of whether they were described in the same or different articles and of whether they shared some individual variants.

#### Manual curation scores

We define confidence scores for an oligogenic combination, first, based on the information presented exclusively in its corresponding publication. These scores carry the ‘Manual’ suffix (Figure 1A, Table 1). A detailed explanation of how each individual strength level for each type of evidence is defined can be found in the Supplementary Data, File 1.

**Table 1.** Summary descriptions of the curation confidence scores linked to the variant combinations present in OLIDA. For each type of evidence, if the information found for a combination does not fulfil the criteria to provide at least a Weak (1) score, an Absent (0) score is assigned. Decision trees (see Supplementary Data, File 1) are used to define the FUNmanual, FUNmeta, FINALmanual and FINALmeta scores. The GENEmanual_harmonized score is defined as the best GENEmanual score assigned for a gene pair among the research articles. More details on how each confidence level is defined per evidence can be found in the Supplementary Data, File 1

FAMmanual: familial evidence based on the article		
Weak (1)	Moderate (2)	Strong (3)
One of two conditions: The genotypic and phenotypic information of only one healthy first-degree relative is described Imperfect segregation in a pedigree with information on two (or more) first-degree relatives	One of two conditions: Information of two (or more) first-degree relatives, showing a perfect segregation of the variants according to the phenotype Imperfect segregation in a pedigree with information on first- and second-degree relatives	Information of healthy first- and second-degree relatives, showing a perfect segregation of the variants according to the phenotype
STATmanual: statistical evidence based on the article		
Weak (1)	Moderate (2)	Strong (3)
Implicit evidence that healthy individuals do not carry the oligogenic combination based on control cohorts or public databases. Known control phenotypes, sufficient control size and matched ethnicity	Explicit evidence that healthy individuals do not carry the oligogenic combination based on control cohorts and public databases. Known control phenotypes, sufficient control size, matched ethnicity and (preferably) similar sequencing technology	NA
STATknowledge: statistical evidence based on databases and cohorts		
Weak (1)	Moderate (2)	Strong (3)
The combination is not found in the 1000 Genomes Project and relevance of all involved variants in ClinVar	NA	NA
STATmeta: maximum of STATmanual and STATknowledge^a^		
Weak (1)	Moderate (2)	Strong (3)
The oligogenic combination is not found in the 1000 Genomes Project. Other implicit evidence of its statistical relevance for the phenotype	The variant combination is not found in the 1000 Genomes Project. Additional explicit evidence that healthy individuals do not carry the oligogenic combination based on control cohorts or public databases of matched ethnicity and sufficient control size	NA
GENEmanual: gene functional evidence based on the article		
Weak (1)	Moderate (2)	Strong (3)
Relevance of involved pathway(s) or expressed tissues on the studied phenotype	One of two conditions: a) Effect of the gene combination on the observed phenotype using a functional experiment with either only a double knock-out or multiple single-gene knockouts b) Direct gene relationship (e.g. common pathway and direct interaction) and relevance for the studied phenotype.	Synergistic or additive effect of the gene combination on the observed phenotype using a functional experiment with single and multiple gene knockouts
GENEknowledge: gene functional evidence based on databases		
Weak (1)	Moderate (2)	Strong (3)
Relevancy of Reactome or KEGG pathways linked with the genes for the observed phenotype.	One of two conditions: a) Gene combination forms a connected PPI network and the comPPI score of each link is >0.8 b) Common Reactome or KEGG pathways, relevant for the observed phenotype.	NA
GENEmeta: maximum of GENEmanual_harmonized and GENEknowledge		
Weak (1)	Moderate (2)	Strong (3)
Relevance of the genes on the studied phenotype using pathway or tissue expression information	Direct gene relationship or effect of the gene combination on the observed phenotype without comparing the individual effects of genes	Synergistic or additive effect of the gene combination on the observed phenotype using a functional experiment with single and multiple gene knockouts
VARmanual: variant functional evidence based on the article		
Weak (1)	Moderate (2)	Strong (3)
One of three conditions: a) All variants are predicted as pathogenic b) Functional experiments for some variants and predicted pathogenic effects for the rest c) Functional experiments using single-variant mutants for the involved variants with a promising but not conclusive effect on the observed phenotype	One of two conditions: a) Effect of the variant combination on the observed phenotype using a functional experiment with either only a double mutant or multiple single mutants b) Clear pathogenic impact of the variant combination on the observed phenotype in an *in silico* analysis of the joint effect of the variants	Synergistic or additive effect of the variant combination on the observed phenotype using a functional experiment with single and multiple gene mutants
VARknowledge: variant functional evidence based on predictors		
Weak (1)	Moderate (2)	Strong (3)
Pathogenicity prediction for all variants by at least one predictor among CADD, SIFT, MutationTester and Polyphen	NA	NA
VARmeta: maximum of VARmanual and VARknowledge		
Weak (1)	Moderate (2)	Strong (3)
Pathogenicity predictions for all involved variants or inconclusive effects of functional experiments	One of two conditions: a) Effect of the oligogenic combination on the observed phenotype using a functional experiment with either a double mutant or multiple single mutants b) Clear pathogenic impact of the oligogenic combination on the observed phenotype in an *in silico* analysis of the joint effect of the variants	Synergistic or additive effect of the variant combination on the observed phenotype using a functional experiment with single and multiple gene mutants
FUNmanual: functional evidence based on GENEmanual and VARmanual FUNmeta: functional evidence based on GENEmeta and VARmeta		
Weak (1)	Moderate (2)	Strong (3)
Based on a decision tree, not enough evidence to suggest synergy, but relevance of the involved genes and variants	Based on a decision tree and evidence of a relationship, as well as potential functional synergy for the involved genes and variants, but the joint pathogenic effect on the studied phenotype is still not confirmed or clear	Based on a decision tree, strong evidence of the functional synergy of both involved genes and variants on the studied phenotype
FINALmanual: overall evidence based only on Manual scores FINALmeta: overall evidence based on Manual and Knowledge scores		
Weak (1)	Moderate (2)	Strong (3)
Based on a decision tree and evidence of the relevance of the variant combination for the observed phenotype but not enough to show that the involved variants are the only culprits for the studied phenotype or that the cause is indeed oligogenic	Based on a decision tree, good genetic and functional evidence of an effect of the oligogenic variant combination on the observed phenotype, but the described information/mechanism is not clear or strong enough to provide proof of oligogenicity	Based on a decision tree and strong evidence of the synergistic/additive effect of the oligogenic variant combination on the observed phenotype genetically and functionally

a Exception: if STATknowledge = 0 (because the variant combination is found in the 1000 Genomes Project), then it replaces STATmanual and, thus, STATmeta is also 0.

The Familial Manual Score (FAMmanual) represents the strength of familial evidence that the variant combination is linked to the observed phenotype by looking at the segregation of variants in the pedigree described in the corresponding publication, which should include both the patient(s) and healthy relatives. The Statistical Manual Score (STATmanual) represents the strength of statistical evidence in the corresponding publication that the variant combination does not occur by chance in an individual with the observed phenotype using either (i) a control cohort of matched ethnicity and sufficient size or (ii) one (or more) of the numerous available databases containing genetic data of individuals (30–32). A STATmanual score of 2 is the maximum score this type of evidence can obtain as, in such studies, the environmental background of the individuals cannot be efficiently controlled compared to pedigrees.

The Gene Combination Manual Score (GENEmanual) represents the strength of evidence shown in the corresponding article for the functional relationship between the involved genes of an oligogenic combination (e.g. biological processes, co-expression and PPIs) and their relevance for the studied disease phenotype (e.g. experiments on animal models and pathways linked with phenotype). This can be shown through experiments *in vivo* or *in vitro* and/or with computational analyses. The Variant Combination Manual Score (VARmanual) represents, on the other hand, the strength of functional evidence presented in the corresponding article for the joint effect of the variants of an oligogenic combination on the observed phenotype *in vivo* (e.g. animal models) and/or *in silico*(e.g. pathogenic effect predictors). Negative results in functional experiments negatively impact these scores. The GENEmanual and VARmanual scores of an oligogenic combination are then combined together using a decision tree (Supplementary Figure 1) in order to define the aggregate Functional Manual Score (FUNmanual), which represents the joint gene and variant functional evidence.

Finally, the FAMmanual, STATmanual and FUNmanual scores are combined together to obtain the Final Manual Score (FINALmanual) for the oligogenic combination by using a decision tree (Supplementary Figure 2). This score represents the overall confidence of the pathogenicity of the oligogenic combination and its link to the observed phenotype based exclusively on the information presented in its corresponding article.

#### Evaluating evidence of shared variants among different combinations

In general, the presence of one or more variants of a combination in another cannot, according to our criteria, provide direct statistical or functional proof that the combination in question is relevant for the studied phenotype. A different variant combination, even with one shared variant, still requires different genetic and functional evidence. Every variant combination evidence is thus unique.

The information of shared variants among combinations is only taken into account for the relevance of the individual variants and is subject to the discretion of the curators as additional proof in the following cases: (i) if this variant is present in controls and never found alone in patients but always in combination with another variant, indicating thus a modifier role, or (ii) if that a variant is found only in patients, alone or with another variant, and is absent in controls, indicating a more dominant role, or (iii) if other patients carrying a variant in one particular gene (in combination with other variants) always have an earlier onset or more severe symptoms than patients not carrying this variant in e.g. a cohort study. This information provides some additional statistical proof of the relevance of a single variant but can only be used in combination with the main part of the statistical proof of absence/presence in controls and based on the information of the other variants in the specific combination that is being curated.

Similarly, for functional evidence, a reference to another article showing the functional effect of a single variant on gene function is considered relevant only when the effect is linked to the studied phenotype.

### Post-curation data processing

After gathering and scoring all oligogenic variant combinations with a Manual Score, the data went through several processing steps to standardize the available information for OLIDA (Supplementary Data, File 1). The disease names were standardized using the Orphanet database (https://www.orpha.net/). The gene names were standardized according to the gene nomenclature guidelines found on the HUGO Gene Nomenclature Committee (HGNC) database (33). The variants were processed by the software Synvar (http://goldorak.hesge.ch/synvar/) to obtain their genomic coordinates. Those that could not be mapped with Synvar were annotated with the help of Varsome (34) and Single Nucleotide Polymorphism Database (dbSNP) (35). The variants for which the genomic coordinates could not be directly obtained (2%) were further annotated with specific flags (Supplementary Data, File 1).

We then performed a harmonization of the Manual scores. To correct the literature bias for gene combinations among the different articles, we created, for each gene combination, a GENEmanual_harmonized score, which represents the highest corresponding GENEmanual score found among the curated articles for that gene combination. Furthermore, for an oligogenic combination described in multiple papers, we assigned the highest Manual score from each type of evidence found among the papers describing that combination and recalculated its FINALmanual score using the decision trees. This harmonization better depicts the fact that different papers may focus their efforts on different aspects of proving the oligogenicity of a combination (e.g. one can focus on the genetic evidence of a genotyped pedigree and another on proving the synergy of the genes and variants with functional experiments).

#### Knowledge curation scores

For certain types of evidence, we created additional scoring measures that compensate for missing information in the corresponding articles (e.g. older articles did not have access to large biobanks or miss experimental evidence appearing later in time) by using public external databases. These scores carry the ‘Knowledge’ suffix (Figure 1B, Table 1) and are assigned only if information is found for all units of the combination involved. Each Knowledge score value has an equal meaning and strength as its corresponding Manual score value. A detailed explanation on how each individual strength level for each type of Knowledge score is defined can be found in the Supplementary Data, File 1.

The Statistical Knowledge Score (STATknowledge) is assigned by checking the presence of a variant combination in the 1000 Genomes project (30) and the citations linked with the involved variants in ClinVar (36) to assess their link with the studied disease (Table 1). The 1000 Genomes project was chosen because it is one of the main public databases giving access to genomes of diverse ancestry. Combinations that are present in the 1000 Genomes project or whose variants do not have a link to the studied disease in ClinVar have a score of STATknowledge score of 0.

The Gene Combination Knowledge Score (GENEknowledge) is assigned by using PPI and pathway information as the rest of the gene-related information used for curation (e.g. co-localization and expression in the same tissue) was deemed as not strong enough to provide an automated score on its own (Table 1). PPI information was obtained with the comPPI database (37), and an interaction confidence score of 0.8 was used as a threshold for accepted interactions. The pathway information was retrieved from Kyoto Encyclopedia of Genes and Genomes (KEGG) (38) and Reactome (39). A manual screening then occurred to verify the presence of common pathways in either KEGG or Reactome and whether these pathways are relevant for the studied phenotype.

The Variant Combination Knowledge Score (VARknowledge) is assigned by using variant pathogenicity information from different pathogenicity predictors: SIFT (40), MutationTaster2 (41), CADD (42) and Polyphen2 (43) (Table 1). These tools were chosen for their easy access, regular updates and wide usage in research papers, as well as the fact that they assess a different impact on the protein function. Due to the fact that DIDA (and consequently part of OLIDA) is used as training data for VarCoPP (19), we did not use VarCoPP as a way to assess the pathogenicity of the variant combinations, thus avoiding any circularity. For CADD, the pathogenicity threshold is defined with the Phred value 15 and, for Polyphen2, both ‘possibly damaging’ and ‘probably damaging’ values are accepted as an indication of deleteriousness.

#### Metascores

Finally, both Manual and Knowledge scores are combined in order to create the confidence Metascores for each type of evidence (Figure 1C, Table 1). These scores represent a more complete way to assess the relevancy of an oligogenic combination as they use information from the corresponding article and public databases. For each oligogenic combination, statistical (STATmeta), gene combination (GENEmeta) and variant combination (VARmeta) metascores are defined as the maximum score found between their corresponding Manual and Knowledge scores. For the GENEmeta, this was obtained as the maximum of the GENEmanual_harmonized and the GENEknowledge. One exception occurs in this rule: if the STATknowledge is 0 due to the fact that the combination is found in an individual of the 1000 Genomes project, then the STATmeta is set to 0.

Once the individual evidence metascores are defined, the same process as in the manual curation is followed in order to define the Final Metascore (FINALmeta) for each combination by using the decision tree (Table 1, Supplementary Figures 1–2). This time, instead of the manual curation scores, the metascores are used for each type of evidence, except for the FAMmanual. First, the gene and variant combination scores are combined to define the aggregated Functional Metascore (FUNmeta), and all information is then used to define the FINALmeta score of a combination.

### Development of the OLIDA database and website

The development of OLIDA consisted of three main aspects. First, a new web portal was built from the ground up with the Django web framework (https://www.djangoproject.com/). The new web portal is in accordance with the FAIR principles and with Django to allow for an improved and more flexible long-term maintenance of the website. This also comes with added security and improved performance for data access. Moreover, an intuitive submission interface was developed to encourage users’ contribution to the database expansion. Finally, a login feature was developed to allow users to keep track of their submissions and remotely access the API.

Second, the database structure is now developed in PostgreSQL (https://www.postgresql.org/), as opposed to the use of MySQL for DIDA. We adapted the database in order to establish the six entities describing the oligogenic variant combination: oligogenic variant combinations, variants, genes, gene combinations, diseases and references. Numerous intermediate tables were introduced to allow many-to-many relationships between the entities, such as the variants and the oligogenic variant combinations or the number of publications linked to an oligogenic variant combination (Supplementary Data, File 1, Supplementary Figure 3).

Finally, a REST API was added with the Django REST framework (https://www.django-rest-framework.org/). The specification of the API follows the OpenAPI (https://swagger.io/specification/) standards.

## Results

OLIDA is now the largest collection of objectively curated and scored data on oligogenic variant combinations involved in genetic diseases. The information on these combinations and the evidence that supports their involvement in disease was obtained by a thorough manual curation of scientific articles, supplemented with information retrieved from public databases.

### General statistics of the oligogenic combinations in OLIDA

The curation of the 262 scientific articles that were obtained via PubMed and passed the relevancy filters (see Materials and Methods) led to the inclusion of 916 oligogenic variant combinations linked to 159 genetic diseases in OLIDA. These combinations involve 1974 distinct variants located in 757 distinct genes. While DIDA was limited to combinations of variants in two genes, OLIDA now also contains 191 combinations with variants in more than two (and up to 17) genes. Additionally, the database now includes 62 combinations involving 31 distinct CNVs, which were not collected in DIDA.

A large proportion of variants in OLIDA is not found in public databases, with 68% and 33% of variants not being reported in the 1000 Genomes Project (1KGP) and gnomAD, respectively. Furthermore, 90% of the variants reported in the 1KGP and 95% of the variants reported in gnomAD have a minor allele frequency of less than 1% in these databases, indicating that the large majority of variants in OLIDA are rare. Annotating the variants using four different pathogenicity predictors (CADD, Polyphen2, SIFT and MutationTaster) resulted in 85% of the variants predicted as disease-causing by at least one of the prediction tools.

**Figure 2.**
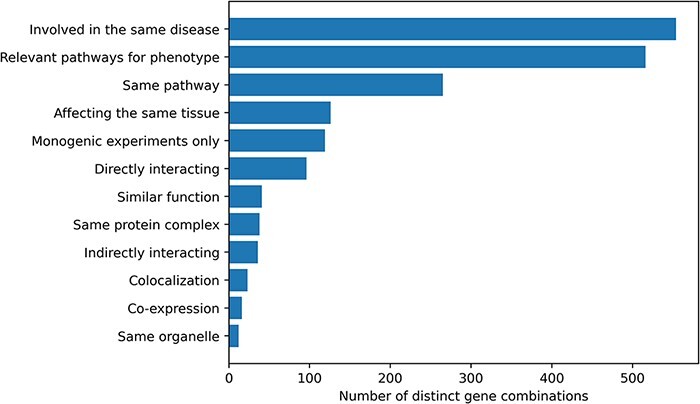
Histogram of the different gene relationship types found between the genes involved in an oligogenic variant combination. The types of gene relationship were obtained either directly from the articles or from public databases (for the ‘Relevant pathways for phenotype’, ‘Same Pathway’ and ‘Directly Interacting’ relationships). Genes are ‘Involved in the same disease’ if patients with the same phenotype described or referenced in the manuscript carried mutations in those genes, together or independently. Pathway information for each gene was either described in the article or found in the KEGG (38) or Reactome (39) databases and was then manually screened to check if the genes belong to ‘Relevant pathways for the phenotype’ (e.g. glucose metabolism pathway for a diabetic phenotype) or in the ‘Same pathway’. Similarly, genes ‘Affecting the same tissue’ must also be expressed in the same relevant tissue for the phenotype. The ‘Directly interacting’ denotes a PPI, either described in the article or retrieved from the comPPI database (37). It is distinguished from the ‘Same protein complex’ relation where the gene products are considered to only fulfil their function when linked together (e.g. the subunits of a channel). ‘Indirectly interacting’ genes are those whose products indirectly interact with an intermediate protein or are involved in a gene regulation mechanism with other gene products (e.g. transcription factors). ‘Similar function’ indicates that genes have the same function (e.g. motor proteins). ‘Co-localization’ implies a direct overlap of the location of the gene products in the cell (e.g. shown using immunofluorescence), while ‘Same organelle’ implies that the protein products exercise their function in the same organelle (e.g. cilia proteins). The ‘Co-expression’ relationship implies a positive correlation of the mRNA expression of the genes in a temporal fashion shown or referenced in the article. Finally the ‘Monogenic experiments only’ notes the fact that the experimental evidence and the assessment of their pathogenicity were done on the genes independently (e.g. single knockouts).

Information about the relationship between the genes involved in an oligogenic combination and their link to the disease was collected during the curation process. Ten different types of gene relationships were identified and assigned to the combinations during both the manual curation process and the post-curation processing (Figure 2). The vast majority of combinations in OLIDA (82%) have genes that are at least known to be involved in the same disease based on information from previous studies or public databases (e.g. ClinVar) or are in pathways that are relevant for the disease phenotype (76%). By focusing only on the terms that represent a biological relationship between genes (e.g. same KEGG or Reactome pathway, expression in the same tissue), out of the 678 distinct gene combinations, 402 (59%) combinations have at least one type of gene relationship, with a certain amount of combinations (30%) having more than one gene relationship type.

There are three times more genetic diseases represented in OLIDA (159) compared to DIDA (52). Over-represented diseases in DIDA included Bardet–Biedl syndrome (20%), familial haemophagocytic lymphohistiocytosis (13%) and familial long QT syndrome (13%). In OLIDA, new diseases appear to be on top of this list and include Kallman syndrome (10%), amyotrophic lateral sclerosis (10%), isolated anencephaly (8%) and normosmic congenital hypogonadotropic hypogonadism (8%). For the case of isolated anencephaly, it is important to note that all the combinations are derived from a single cohort published in a research article (44). OLIDA also includes diseases newly linked with oligogenic signatures, such as arthrogryposis syndrome, holoprosencephaly, adolescent idiopathic scoliosis and Müllerian aplasia. More than half of the diseases in OLIDA (59%) are linked with only one or two associated oligogenic combinations.

It is important to note that, in addition to collecting combinations involving more than two genes and combinations involving CNVs, OLIDA also gathers more information on the variant combinations that were already present in DIDA. In particular, it collects statistical evidence on the variants by checking their presence in large databases for both positive and negative evidence of their involvement in disease, as well as new information on the relationships between the genes involved in the combinations, with the addition of new terms such as ‘Same organelle’, ‘Involved in the same disease’ and ‘Relevant pathways for phenotype’. One example where such extra information is added is the combination OLI302 in OLIDA, which corresponds to the combination dd021 in DIDA. This particular instance was described to have both familial and functional evidence in DIDA, and OLIDA now quantifies this evidence showing that it was supported by weak familial (FAMmanual score of 1) but strong functional evidence (FUNmeta scores of 3), thus indicating a synergistic effect of the variants involved. Moreover, OLIDA points out that this combination is also supported by weak statistical evidence (STATmeta score of 1). Finally, the new curation process includes new types of gene relationship terms that are directly obtained from the articles, such as the ‘Relevant pathways for phenotype’ term attributed to this combination. Another example is the combination dd002 in DIDA, which corresponds to the combination OLI192 in OLIDA. This combination is not linked with any functional evidence in DIDA but has a moderate FUNmeta score of 2 in OLIDA due to the integration of new sources of information during the post-curation process. The variants involved in this combination were predicted as pathogenic by pathogenicity predictors during the annotation process for the VARknowledge score, which were probably not available at the time the article was written and were therefore not referenced as part of functional evidence in DIDA. Here again, the gene pair is also associated with additional gene relationship terms compared to DIDA, such as ‘Same protein complex’ and ‘Affecting the same tissue’.

### Statistics on the pathogenicity confidence levels of the oligogenic variant combinations

In order to depict the confidence of disease causality for each oligogenic combination based on the available information, we established a transparent and thorough curation protocol that provides confidence scores for different types of evidence linked with the pathogenicity of an oligogenic combination (familial, FAM; statistical, STAT; gene combination functional, GENE; variant combination functional, VAR; see Materials and Methods, Figure 1, Table 1, Supplementary Data, File 1). These individual scores were then combined to obtain a final confidence score (FINAL, Supplementary Figures 1**–**2) for each oligogenic combination, which depicts the overall confidence of its association to disease, based on the premise that a combination ideally requires both adequate genetic and functional evidence to be considered as causative. The scores were obtained through the manual curation of the corresponding articles (‘manual’ suffix, Figure 1A), through information from public databases (‘knowledge’ suffix, Figure 1B) and, finally, through a combination of the article and database information (‘meta’ suffix, Figure 1C) for a more holistic assessment of their association to disease. With this process, we, therefore, created a comprehensive repository of the oligogenic variant combinations reported in the literature as linked to a disease phenotype and provide a basis to assess these combinations according to the level of evidence that associates them to disease.

**Figure 3.**
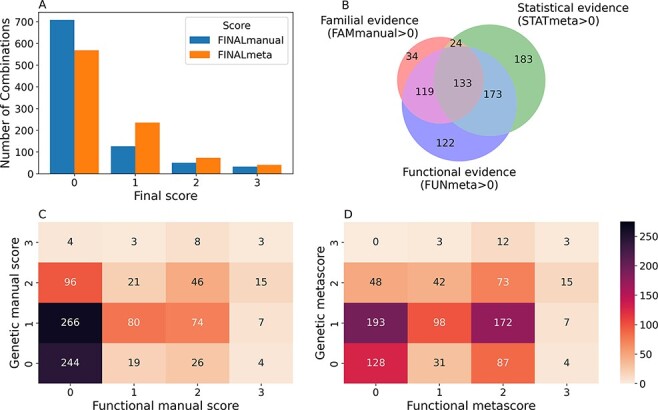
Confidence scores and types of evidence present in the OLIDA combinations. (A) Distribution of the FINALmanual and FINALmeta scores. (B) Venn diagram of the number of oligogenic combinations carrying a score of 1 or higher in the different main types of evidence metascores. The 130 oligogenic combinations whose FAMmanual, STATmeta and FUNmeta scores are all 0 are not shown in this diagram. (C) Heatmap of the number of combinations and their confidence functional and genetic scores based on the evidence collected via manual curation (Manual scores) only and (D) when adjusted using the external database information (Meta scores). The genetic score here represents the maximum score between the FAMmanual and STAT (manual or meta for plots a and b, respectively) and the functional score is the FUN (manual or meta for plots a and b, respectively), which are described in the Materials and Methods.

From the 916 combinations present in OLIDA, 208 (23%) initially had a FINALmanual confidence score larger than 0 (i.e. based on information present exclusively in their corresponding article). The addition of information from external databases to supplement any missing information from the articles resulted in 348 (38%) oligogenic combinations with a FINALmeta of 1 or higher (Figure 3A). The majority of these combinations have a FINALmeta score of 1, being linked with at least the minimum amount of evidence (according to our curation criteria) of association to the genetic disease. From all combinations in OLIDA, 133 combinations are linked with all three types of evidence for oligogenicity (familial, statistical and functional), while 119 carry familial and functional evidence and 173 carry statistical and functional evidence (Figure 3B). Only three combinations, all digenic, are associated with strong oligogenicity confidence (score of 3) for both genetic and functional evidence: OLI111, OLI179, OLI200, associated with Charcot-Marie-Tooth disease (45), cystinuria (46) and familial isolated hypertrophic cardiomyopathy (47), respectively. The common denominator among all these three cases is that they provide strong familial evidence of the segregation of variants in large pedigrees with first- and second-degree relatives and support with functional experiments the pathogenic joint effect of the genes and variants on the studied phenotype compared to their individual effects. On the other hand, 73 oligogenic combinations are associated with moderate evidence (score of 2) for both genetic and functional evidence (Figure 3D).

The oligogenic combinations involving more than two genes, which were absent in DIDA, overall present lower confidence scores than the digenic ones. The large majority (85.3%) of these combinations have a FINALmeta score equal to 0, with 24 (12.5%), 2 (1%) and 2 (1%) combinations being assigned a score of 1, 2 and 3, respectively. The distribution of FINALmeta scores for these combinations is more skewed than the one for the combinations with variants in two genes only, where 405 (56%) combinations have a FINALmeta score of 0, 211 (29.1%) of 1, 71 (9.8%) of 2 and 38 (5.2%) of 3.

A big contributor to the fact that the majority of combinations have a FINALmeta score of 0, especially for those combinations involving more than two genes, is the lack of available functional evidence either in their manual (Figure 3C) or meta scores (Figure 3B, D). In total, 371 (40%) combinations are associated with a FUNmeta score of 0, from which 130 do not carry any sufficient genetic evidence (Figure 3D), while 241 combinations are linked exclusively with genetic evidence: 183 are linked exclusively with statistical evidence, 34 only with familial evidence and 24 with both familial and statistical evidence (Figure 3B). On the other hand, 120 (13%) oligogenic combinations are associated only with functional evidence (Figure 3B).

An example of an oligogenic combination involving variants in more than two genes with strong confidence in its association to the disease is OLI606 (48). This combination involves three heterozygous variants in the genes *ΜΥΗ7, MRTFB (MKL2* in the publication) and *NKX2-5* identified in a family with left ventricular noncompaction (LVNC), with a suggested Monogenic plus modifier oligogenic effect. In the family, while the children carrying all three variants presented with early onset LVNC, first- and second-degree relatives carrying a variant in either *NKX2-5* or *MRTFB* were healthy, whereas the father carrying a combination of the variants in *MRTFB* and *MYH7* was asymptomatic. The effect of the *de novo* variant in the *MYH7* gene alone was not shown. Therefore, as the segregation in the family involved both first- and second-degree relatives, but was not shown as complete, even if the *MYH7* variant alone most probably does not infer early onset LVNC based on the information described in the pedigree, we assigned a moderate FAMmanual score. These variants were then studied using an *in vivo* mouse model showing that the mice harbouring the three heterozygous variants had a significantly reduced cardiac function when compared to double-mutant, single-mutant and wild-type mice. Based on the evidence of synergy among the genes and variants for that combination, both GENEmeta and VARmeta scores are strong. The combination of both familial evidence and functional evidence in this article led to the attribution of a FINALmeta score of 3 to this trigenic combination, showing that there is strong genetic and functional proof of the synergistic effect of the involved variants.

Another interesting example of a combination with less evident proof is the oligogenic combination OLI474, associated with congenital hypothyroidism, derived from a cohort study and involving three heterozygous variants in the genes *DUOX2, TG* and *TPO* (49). The study did not provide sufficient information, according to our criteria, for familial evidence as the phenotypes of the parents and their genotypes were not clearly described. However, statistical genetic evidence was shown for each individual variant using a cohort of 100 individuals of matched ethnicity with the patients; all variants were relevant in ClinVar, and this combination was also not found in the 1000 Genomes Project, leading to a weak STATmeta score. The three genes are referenced to be involved in the same biological process of thyroid hormone synthesis without further experiments on synergy for the observed phenotype, and, therefore, the FUNmeta score is moderate. On the other hand, although one variant was shown to be benign in this combination based on the available information in the paper, the VARknowledge score is weak, as all variants had at least one pathogenic prediction, and therefore the VARmeta score for this combination is weak. The combination of both genetic and functional evidence led to a weak FINALmeta score, showing that although some evidence of oligogenicity exists, the genetic and functional evidence for synergy among genes and variants is not strong enough for a more confident conclusion.

### Description of OLIDA

**Figure 4.**
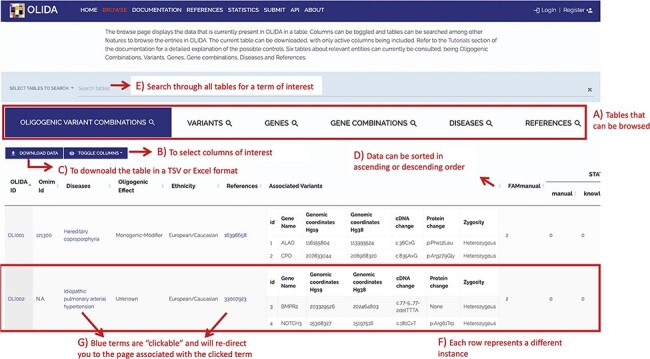
Screenshot of the Browse page of OLIDA with the Oligogenic Variant Combinations selected showing the different possibilities that the database offers. Six different tables can be browsed (A) with the currently selected one shown in blue. (B) The user can then select the columns of interest to be displayed in the table and (C) download the table with the selected columns. (D) In a particular table, data can be sorted in ascending or descending order based on a particular column’s data. (E) A specific term (e.g. gene name and disease name) can be used to search all tables. (F) Each row represents a specific instance and (G) clicking on specific terms in blue will bring the user to the detail page for that specific instance.

**Figure 5.**
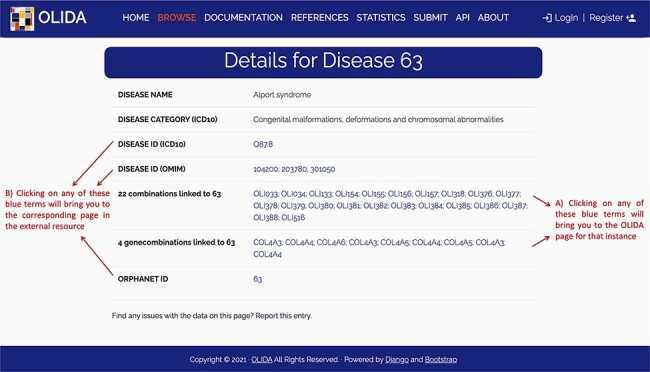
Screenshot of the detailed page for Alport syndrome. This page allows the user to visualize in more detail any instance of the database. It provides (A) links between this instance and the other entities of the database, as well as (B) clickable links towards corresponding pages in external databases where information about this entity was retrieved.

The information in OLIDA is organized in different tables corresponding to the different entities that are involved in an oligogenic variant combination: oligogenic variant combinations, variants, genes, gene combinations, diseases and references. These six different tables can be accessed through the ‘Browse’ page of the website (Figure 4A). Each table collects, in specific columns, different types of data on the instances, such as associations with other entities of the database (e.g. associated genes and variants for oligogenic variant combinations), or annotations from external tools and databases (e.g. pathogenic predictions for variants or different gene identifiers for each gene). The content of each column is described in the documentation of the website, and the columns of interest can be selected to be shown or hidden using the ‘toggle’ function (Figure 4B). The full tables with the selected columns can be downloaded directly in the browse page (Figure 4C), with the data sorted in ascending or descending order of a particular column (Figure 4D) according to what was pre-selected by the user. Each line in a table represents a different instance. Particularly for the oligogenic combinations table, since oligogenic variant combinations can have variants in any number of genes, the variants that are associated with an oligogenic combination are represented by a sub-table in which each line represents a variant involved in the combination. Each instance also has a specific page, which can be reached by clicking on the identifier of the instance (Figure 4F), and provides more details on the variant combination, variant, gene, gene combination, disease or reference of interest (Figure 5). Finally, blue terms in each line are clickable (Figure 4G) and will redirect the user to the detailed page for the particular instance linked to that term (if it is an OLIDA entity) or to the external resource this term refers to (e.g. clicking on the rsid of a variant in the variants table will bring the user to the corresponding variant page in dbSNP).

### Searching and accessing the information in OLIDA

A general search can be done by entering specific search terms (Figure 4E), which will dynamically look for that term in the six tables contained in OLIDA (e.g. the cDNA of a specific variant such as c.203C > T, a specific gene name like *ALAD*, or search directly for an oligogenic combination, like OLI111). The results containing only this particular search term will be shown in all six tables. Alternatively, the information contained in the database can also be accessed through an API, with two potential interfaces: a Swagger-UI (https://olida.ibsquare.be/api/swagger/) or Redoc (https://olida.ibsquare.be/api/redoc/) interface. All tables can also be downloaded in TSV or excel format directly from the browse page.

### Submitting new oligogenic combinations

OLIDA encourages users’ contributions to the collection of data on oligogenic diseases by providing a ‘Submit’ page, where users can send information on variant combinations linked to an oligogenic disease. The user must at least provide information about the related publication and the oligogenic variant combination themselves, with the possibility to choose existing combinations from the database or create new ones. These submissions will then be verified and further annotated by curators, before getting integrated into the database.

Tutorials on how to navigate and download the data, as well as more details on how to submit new data are available at https://olida.ibsquare.be/documentation/

## Discussion

OLIDA is now the largest comprehensive collection of curated and scored information on oligogenic variant combinations linked to human diseases, going substantially beyond DIDA (18). Although the study of oligogenic diseases is still in its infancy, DIDA itself has led to the development of methods that aim to understand and predict the genetic architecture of digenic diseases. The creation of OLIDA that now moves further in the genetic disease continuum is an important step towards a better understanding of the causes of oligogenic diseases providing high-quality information. It furthermore opens the discussion for the establishment of improved standards in this field as this became apparent from the creation of the detailed curation protocol presented in this work.

OLIDA required several novel developments at the technical level. Collecting information on combinations in any number of genes required a complete redesign of the database schema and website, in particular with regard to the relations between the tables linking the variant combinations to the genes and variants, as well as the information reported in the website pages. Moreover, OLIDA expands the variant information beyond the subset of small variants (e.g. SNPs and indels), including combinations involving structural variants and CNVs, which have also been reported to be involved in oligogenic diseases, such as in amyotrophic lateral sclerosis (27). As it seems that the existing standard for CNVs description is not closely followed in the scientific literature, we created our own representation for such instances in the database. Additionally, the number of studies on oligogenic variant combinations has significantly increased, and overlaps have occurred between articles since the creation of DIDA, highlighting the need to be able to not only associate a publication to several variant combinations but also link specific oligogenic combinations to several publications. Furthermore, OLIDA now closely follows the FAIR principles on data management (29). With the indexing of the pages in search engines, the existence of an API, the open availability of the data, the use of unique identifiers and links to existing ontologies, as well as an explicit data licence, OLIDA now contributes to open science and the study of oligogenic diseases in the most possible FAIR way.

An important innovation provided by OLIDA is that it is based on a transparent curation process that assigns confidence scores for each oligogenic combination. These scores are created using structured and clearly defined criteria reflecting the level of evidence supporting the causality of the combination for its associated disease. This evidence is obtained, first, based on the associated article information searched by at least 2 independent curators, and, second, by exploring the current knowledge present in public databases, in order to obtain the final evidence metascores that holistically reflect the availability of information from both sources (Figure 1). The confidence metascores can be particularly helpful in allowing the user to assess how confidently a particular combination found in OLIDA is actually linked with its associated disease based on the existence of adequate genetic and functional evidence. Since the number of publications identifying oligogenic causes to disease is increasing, the establishment of clear specialized standards, such as the ones described in our protocol, required to identify a variant combination as causative of a particular disease is becoming essential.

We assigned 348 out of 916 (38%) oligogenic combinations with a FINALmeta score of 1 or higher, meaning that these combinations have the minimum required genetic and functional evidence—according to our criteria—to make them at least relevant to their associated disease. The fact that the majority of oligogenic combinations present in OLIDA are not assigned a confidence score above 0 can be attributed in most cases to the absence of sufficient functional evidence—missing in 371 (40%) of the combinations—since the description of the functional synergistic consequences of the involved variants on the disease phenotype and the role of the genes they are located in is lacking or unclear (Figure 3D). This is an important aspect of our curation protocol, as it is based on the premise that both genetic and functional evidence showing the synergistic effect of the involved variants and genes should be present to prove causality for a particular disease (28). The lack of such evidence can be mostly explained by the fact that the vast majority of articles in OLIDA are cohort studies or reports on clinical cases, and, thus, the authors of these articles were mostly focused on obtaining genetic evidence on the variant combinations. Furthermore, during the manual curation, we observed discrepancies in the functional evidence among the different articles describing similar gene combinations as some lacked certain methodologies that were not available at the time of writing, such as the use of variant pathogenicity predictors. Moreover, functional knowledge on gene relationships builds up over time as, for example, most Bardet–Biedl syndrome genes are now known to be involved in the same protein complex (50), whereas this was not the case when the first articles suggesting oligogenic inheritance in this disease were published. This observation motivated our implementation of knowledge and metascores for the combinations, which helped to objectively increase the functional scores of a significant number of instances. Nevertheless, it is evident that important functional knowledge on the synergistic mechanisms among genes, even for those previously reported to be involved in the same disease, is still missing. As we are opening the discussion about new standards in the reporting of oligogenic combinations, we hope that this limitation in functional evidence will be addressed in future studies reporting oligogenic cases.

We acknowledge that the criteria used to attribute the confidence scores could introduce a bias towards more closely related genes as, for example, a moderate (2) GENEmeta score is assigned to combinations whose genes are involved in the same pathway or are directly interacting without other clear synergistic experiments for the studied phenotype and a weak (1) GENEmeta score is assigned to combinations whose genes are involved in different, but relevant for the phenotype, pathways, without further functional evidence. Nevertheless, it is important to note that this choice of scoring does not imply that only genes that are biologically very closely related can be involved in oligogenic diseases. Indeed, phenomena such as indirect epistasis (i.e. genes being involved in different pathways but that could impact general important metabolic processes, such as signalling or developmental pathways) show that understanding the biological mechanisms behind gene interactions is a complex problem. This choice of scoring rather depicts the fact that the functional evidence described for genes that are more closely related is usually more direct and clearer compared to the evidence for genes suggested to be involved in indirect epistasis. For the latter, in most cases, additional functional analyses need to be conducted to demonstrate and clarify the epistatic mechanisms involved and, if this is the case, as shown with our curation criteria, the GENEmeta score can be strong (3). This observation further depicts the need for improved functional assays to detect epistasis for such more complex cases.

The curation process is, at the moment, semi-automatic, which can present certain limitations. Although the annotation and attribution of the knowledge scores are mostly automated, the initial extraction of information is done by curators who must read and discuss each article. Certain annotation parts also require manual input, such as the processing of variant coordinates, for which information is often missing, leading to an overall time-consuming curation process. We are currently working on developing data mining tools specific to the collection of data on oligogenic diseases in order to decrease the time spent extracting the information from articles.

We encourage the authors of articles describing oligogenic variant combinations to contribute to our effort by submitting their data through the database submission system. The increase of data and the involvement of the scientific community in their assessment are crucial to advance our knowledge on the synergistic mechanisms and the genetic components behind oligogenic diseases.

## Supplementary Material

baac023_Supp

## Data Availability

The OLIDA database is available online under a Creative Commons Attribution-NonCommercial 4.0 International License at https://olida.ibsquare.be and via its REST API (https://olida.ibsquare.be/api/swagger/ or https://olida.ibsquare.be/api/redoc/). The data can be downloaded as TSV or EXCEL data files.
