# Supplementary material for: Scaling up oligogenic diseases research with OLIDA: the Oligogenic Diseases Database
Source: Database (Oxford). 2022 Apr 12;2022:baac023. doi: 10.1093/database/baac023 (PMC9216476; doi:10.1093/database/baac023)
Supplement: baac023_Supp [file baac023_supp.zip › Supplementary_File ...doc]

# Supplementary File for

# Scaling up oligogenic diseases research with OLIDA: the Oligogenic Diseases Database

Charlotte Nachtegael^1,2^*, Barbara Gravel^1,2,3^*, Arnau Dillen^3,4^, Guillaume Smits^1,5,6^, Ann Nowé^1,3^, Sofia Papadimitriou^1,2,3^** and Tom Lenaerts^1,2,3^

* Joint first authors

** Corresponding author

# Content

Supplementary Methods

OLIDA database structure

Development of the OLIDA website

Manual curation procedure

Evaluating evidence of shared variants among different combinations

Post-curation data processing

Metascores

Supplementary Figures

Supplementary Figure 1

Supplementary Figure 2

Supplementary Bibliography

# Supplementary Methods

## OLIDA database structure

The database is built around six entities: the oligogenic variant combinations, its components which are the variants, the genes, the gene combinations and the diseases, as well as the references which were curated to extract the variant combinations. Each table contains properties and attributes attached to the entity, gathered during the curation and the post-curation process by automatic annotations with several biological databases and tools.

In order to upgrade from the digenic variant combination in DIDA to the oligogenic variant combination in OLIDA, we created an intermediate table (VariantCombination) to allow a many-to-many relationship between the variant combinations and the variants and determine their zygosity in that particular oligogenic variant combination, as well as between the variant combination and the diseases (DiseaseCombination) to include the Dual Molecular Diagnosis and syndromic cases. Additionally, we introduced the gene combination entity, representing the information known about the relationships between the genes involved in an oligogenic variant combination. Finally, we allowed many-to-many relationships between the references and the variant combinations (ReferenceCombination) as one reference can have information about several variant combinations, and some variant combinations were found in several articles during our curation process.

We also introduce the Copy Number Variation, in addition to the Small Nucleotide Polymorphisms and Insertion/Deletion variants already present in DIDA referenced here as Small Variants. Copy Number Variations are characterized by their type, the location on the chromosome, their eventual repeated sequence and the lower and upper number of repeats.

The central concept in OLIDA is an oligogenic variant combination, found in at least one patient in an article, arguably causing at least one disease, is composed of several variants and a gene combination. Each oligogenic variant combination is supported by the curation (manual scores) and the knowledge from databases (knowledge scores) computed together as the metascores.

## Development of the OLIDA website

The development of OLIDA consisted of three main aspects. First, a new web portal was built from the ground up with the Django web framework (https://www.djangoproject.com/) for the Python programming language. Second, the database structure was updated and migrated from MySQL to PostgreSQL. Finally, a REST API was added with open data access standards. The new web portal was built according to the FAIR principles.

The web portal was built in Django to allow for an improved and more flexible long-term maintenance of the website. This also comes with added security and improved performance for data access. We also took the opportunity to update the interface of the web portal to bring it more in line with modern web design standards. This also allowed us to use Django’s built-in admin interface to make a custom curator interface and add user profiles. In combination with a new submission procedure, this should form a good foundation to make OLIDA into a community-curated data repository.

To allow for oligogenic combinations of genetic variants, the database schema had to be modified. A new schema design that was previously discussed was created to support this, together with other changes that allow for more flexibility to extend the design. Next to the updated schema, the database backend was migrated from MySQL to PostgreSQL. This should also provide improved performance for data access and facilitate data management more and also unifies the database engine for our other web services.

The newly created web portal for OLIDA was built to make it Findable, Accessible, Interoperable, and Reusable (FAIR)(1). To make it more findable, metadata was added to the site pages to ensure that keywords such as *oligogenic* would bring users to OLIDA upon search queries through a search engine.

To improve accessibility, the new web portal allows downloading the data in multiple formats and exploration of the data in different ways. This also contributes towards making OLIDA interoperable and ensuring that the data is reusable. The newly added REST API also aims to ensure this. The REST API was built using the Django REST Framework (https://www.django-rest-framework.org/) software library. The specification of the API follows the OpenAPI (https://swagger.io/specification/) standards. The structure of returned data is in JSON and formatted according to the JSONAPI (https://jsonapi.org/) standard. The API can be explored through a Swagger-UI (https://olida.ibsquare.be/api/swagger/) or Redoc (https://olida.ibsquare.be/api/redoc/) interface. To maximize interoperability, extensive metadata describes the different items and their relationships.

For detailed documentation on the usage and structure of the web portal, see the documentation at: https://olida.ibsquare.be/documentation/

## Manual curation procedure

### Extraction of papers

Articles were selected using the keywords “digenic OR oligogenic” in PubMed (https://pubmed.ncbi.nlm.nih.gov/), and were further analysed with the help of the abstracts and the text, discarding the studies: a) not involving humans, b) not providing information about the exact variants involved, c) conducting statistics only at the gene level (e.g. gene burden analyses), d) containing chromosomal rearrangements and other large CNVs that span among multiple genes.

From a total of 1501 papers that were initially found in PubMed using these keywords, 262 papers passed these criteria and were used for the curation process.

For each paper, we gathered general information (authors, journal and impact factor), as well as information about the disease and the patients (gender and ethnicity). For the latter case, this information can be useful for later analyses, as some genetic variation could be relevant only to a specific population, while gender can play a role for the analysis of X-linked disorders. We then collected information relevant to the assignment of the confidence scores. We also specified, at each step, the passage in the text containing the referred information, in order to build a resource for eventual future text mining tasks.

Two different curators were assigned per paper. Each curator independently a) read the article, b) extracted the relevant information for OLIDA and c) assigned curation scores to the article. A discussion then took place between them to reach a consensus, in cases when differences arose, either on the extracted information or the curation scores.

All oligogenic variant combinations were evaluated separately, regardless of whether they were described in the same or different papers. The google form that was used to enter the data during the curation process is available at https://forms.gle/7PUfGDU7W5Q6j3679.

### Manual curation scores

Based on the aforementioned premises, we defined confidence scores for each type of evidence, first, based on the information presented exclusively on the publication that describes an oligogenic combination. These scores have the “Manual Score” suffix.

#### Genetic evidence - Familial Manual Score (FAMmanual):

The best type of genetic evidence can be obtained when studying a large pedigree, where the background environment of the involved individuals is controlled as much as possible and a clearer way of studying the segregation of the suspected variants for the observed phenotype is offered. We define the Familial Manual Score (FAMmanual) as:

| FAMmanual = | Strong (3), if the phenotypic and genotypic information for healthy/asymptomatic first (parents and siblings, if available) and second degree relatives is clearly described in the paper, showing a perfect segregation of the variants according to the phenotype.  Moderate (2), if one of two conditions is met:   a) the genotypic and phenotypic information only of first degree relatives (healthy parents, or one healthy parent and at least one healthy sibling) is clearly described in the paper, showing a perfect segregation of the variants according to the phenotype b) the genotypic and phenotypic information of healthy/asymptomatic first and second degree relatives is clearly described in the paper, but the segregation of the variants is imperfect   Weak (1), if one of the two conditions is met:   a) if a perfect segregation of the variants according to the phenotype is not observed in the paper or only the genotypic and phenotypic information of one healthy first degree relative (parent or sibling) is described in the paper b) the genotypic and phenotypic information only of first degree relatives is clearly described in the paper, but the segregation of the variants is imperfect   Absent (0), if no adequate genotypic information is described in the paper for the pedigree or the evidence is not sufficient enough to provide a score of 1 |
| --- | --- |

Some notes on used terms:

- As “perfect segregation” we define the situation where we have information on the phenotypes of individuals in the family that carry the combination of variants in question, each involved variant alone, as well as all possible sub-combinations of the involved variants (in the case of an oligogenic variant combination involving more than two variants).
- For defining the “healthy” individuals, we take into consideration the fact that the oligogenic combinations can have different effects on the phenotype, the main two being a) the phenotype is only observed when the specific combination of variants is present in an individual and b) the “monogenic plus modifiers” scenario, where one variant can have an effect of the phenotype, which is then modulated by the presence of the extra variant(s)(2,3). In the first scenario, healthy individuals are considered as the ones without any disease phenotype, while in the second scenario, we also accept as healthy individuals, those with a less severe phenotype or whose age of onset of disease is later than the individual carrying the oligogenic variant combination.

#### Genetic evidence - Statistical Manual Score (STATmanual)

The second case of genetic evidence is based on a statistical study by either using a) a cohort formed specifically for the study or b) one (or more) of the numerous available databases containing genetic data of individuals(4–6). An important criteria of the strength of the statistical evidence is to compare the individuals of interest with ethnically matched unrelated healthy individuals in an equal or larger quantity than the patients.

We define the Statistical Manual Score (STATmanual) as:

| STATmanual = | Moderate (2), if we have statistical proof of oligogenicity, i.e. healthy individuals can carry either or some of the variants, but not the complete variant combination. For either the cohort or the public databases, (a) the phenotypes of the control individuals should be known, (b) the control size should be at least equal to the size of the patient cohort, (c) the control individuals should be ethnically matched with the patients and (d) *preferably* sequenced with the same sequencing technology. If public databases are used, the authors should elaborate on the statistical comparison and not use them only as a variant pre-filtering step.  Weak (1), if we have at least implicit evidence that controls do not carry this combination or any variants in those genes. In this case, frequency information on gnomAD(5,7), ExAC(7) or other public databases can be used. For either the cohort or the public databases (a) the control size should be at least equal to the size of the patient cohort, (b) the control individuals should be ethnically matched with the patients and (c) *preferably* sequenced with the same sequencing technology. If public databases are used, the authors should elaborate on the statistical comparison and not use them only as a variant pre-filtering step.  Absent (0), if the controls are not at least of equal number as the patients or they are not ethnically matched or incomplete information is available (i.e. information is available for only one variant or no clear consequence is shown for presence of single variants in the control population. |
| --- | --- |

A “healthy” individual is defined in the same way as in the familial evidence.

#### Functional evidence - Gene combination Manual Score (GENEmanual)

The gene combination functional evidence gathers referenced or experimental evidence showing a) a functional relationship between the genes involved in the oligogenic variant combination and b) a relevance for the studied disease phenotype. The functional relationship gives information on whether the genes are involved in the same pathway or different pathways related to the same biological process, are being co-expressed together, have their gene products directly or indirectly interacting, are co-localised in the cell or are expressed in the same tissue. A relevance, on the other hand, gives information on whether the involved biological processes and the tissues these genes are expressed in are relevant for the studied phenotype. A strong gene combination functional evidence should show a synergy between the genes and an effect on the studied phenotype when these genes are not functional, which is not present or is different when only either single gene is not functional.

We define the Gene combination Manual Score (GENEmanual) as:

| GENEmanual = | Strong (3), if a functional experiment *in vivo* or *in vitro* with single and multiple gene knock-outs is described or referenced in the paper and we observe a synergistic or additive effect of the gene combination on the studied phenotype of the organism model carrying the multiple gene knock-outs. This means that both the gene combination effect and their relevance with the studied phenotype is clearly shown.  Moderate (2), if either one of the two following conditions is met:   a) A functional experiment *in vivo* or *in vitro* using either the multiple gene knock-out or the single gene knock-outs is described or referenced in the paper, still observing an effect on the studied phenotype of the organism model(s) carrying the gene knock-outs. b) Information about a direct gene relationship (common pathway, different pathways involved in the same biological process, direct interaction or formation of a complex, co-localisation, expression in the same tissue) and relevance of this information to the studied phenotype is described in the paper for all the genes involved. For the gene relationship, a common pathway or direct interaction/complex formation alone is usually enough to assign this score, while for the rest (co-localisation, expression in the same tissue), we require a combination of at least two different relationship types.   Weak (1), if only the pathway names or tissue expression for the involved genes are described in the paper, without further elaboration on their biological relationship, and this information is relevant for the studied phenotype.  Absent (0), if there is no adequate information of functional evidence for all genes in the combination. |
| --- | --- |

Here, it should be noted that if a clear effect is shown for loss-of-function variants, either those present in the studied combination or for different variants at the same genes, this is also translated as an effect at the gene level. For example, if one of the single variants in the oligogenic combination or a different single variant referenced in the paper leads to a single gene knockout that is linked to a clear effect on the studied phenotype, this effect is also translated at the gene level as gene knock-out evidence, which will also be used to assign the gene combination manual score.

#### Functional evidence - Variant combination Manual Score (VARmanual)

The variant combination functional evidence shows the effect of the combination of variants on the studied phenotype. This type of evidence is separate from the gene combination evidence, as not all variants can have a pathogenic effect, even if there is a proof of functional synergy between the genes of a combination. A strong variant combination evidence should show a synergistic/additive effect when variants occur together compared to when they occur alone. The variant combination functional evidence is divided here in experimental evidence (i.e. *in vitro* and *in vivo* experiments described or referenced in the corresponding paper) and *in silico* evidence (e.g. pathogenic predictors, protein sequence analysis or predictions of disruption of the protein structure).

We define the Variant combination Manual Score (VARmanual) as:

| VARmanual = | Strong (3), if a functional experiment *in vivo* or *in vitro* with single and multiple mutants is described or referenced in the paper and we observe a synergistic or additive effect on the studied phenotype of the organism model carrying the multiple mutants.  Moderate (2), if either one of the two following conditions is met:   a) Only a functional experiment *in vivo* or *in vitro* using either single or multiple mutants is described or referenced in the paper, while still observing an effect on the studied phenotype of the organism model carrying the mutant(s). b) An *in silico* analysis on the joint effect of the variants is described in the paper compared to the effect of single variants, showing a clear pathogenic impact of the variant combination on the function of the gene products and the studied phenotype (e.g. 3D modelling of the protein structure showing the joint disruptive effect of the variants, or variant combination pathogenicity predictors),   Weak (1), if either one of the three following conditions is met:   a) All variants of the combination are predicted as pathogenic by at least one monogenic variant effect predictor (e.g. CADD, Polyphen, SIFT) according to the paper. b) The paper describes or references functional experiments *in vivo* or *in vitro* with single mutants using only some of the involved variants and pathogenic predictions for the rest, with pathogenic effects for all. c) The paper describes or references functional experiments *in vivo* or *in vitro* using single mutants for all involved variants, but the effect on the function of the gene products and the phenotype is not clear or sufficient to put a moderate score.   Absent (0), if there is no adequate information of functional evidence for all variants in the combination |
| --- | --- |

#### Functional evidence - The Aggregated Functional Manual Score (FUNmanual)

The gene combination evidence and the variant combination evidence scores are then combined to obtain the aggregated functional evidence score. For this process, we put more importance on the variant level type of evidence, as it is more precise and specific to the studied oligogenic variant combination. A decision tree (Supplementary Figure 1) was elaborated to decide the final functional score (FUNScore), whose strength and score is defined as:

| FUNmanual = | Strong (3), if there is strong evidence of the functional synergy among all involved genes and variants of the combination, and of their pathogenic effect on the studied phenotype.  Moderate (2), if there is evidence of the functional synergy or relationship among all involved genes and variants of the combination, and of their pathogenic effect on the studied phenotype, but the mechanism is still not clear or strong enough to strongly support oligogenicity.  Weak (1), if there is not enough evidence to suggest synergy, although we can observe evidence of monogenic effects of the involved genes and variants of the combination.  Absent (0), when we do not observe a pathogenic effect or relevant evidence for all units in the oligogenic variant combination (gene or variant) either alone or in combination with other units. |
| --- | --- |

#### The final manual curation confidence score (FINALmanual)

Finally, the familial, statistical and functional evidence is combined together to obtain the final manual curation confidence score (FINALmanual) for the oligogenic combination, using a decision tree (Supplementary Figure 2). The strength of each FINALmanual scores is defined below:

| FINALmanual = | Strong (3), if we have a strong genetic evidence and proof of functional synergy among all involved genes and variants of the oligogenic combination, with a clear pathogenic synergistic effect on the studied phenotype.  Moderate (2), if we observe a good genetic segregation and functional synergy among all involved genes and variants of the oligogenic combination, showing an effect on the studied phenotype, but the described mechanism is still not clear or strong enough to provide proof of oligogenicity.  Weak (1), if the presented information on the oligogenic combination fulfills the basic requirements of a minimum presence of genetic and functional evidence to suggest that there is a pathogenic effect on the studied phenotype and that the variants do not occur by chance, but either there is no evidence of synergy or we are not certain that these are the only culprits for the studied phenotype.  Absent (0), for the rest of the oligogenic combinations that do not fulfill any of the aforementioned conditions. |
| --- | --- |

## Evaluating evidence of shared variants among different combinations

In general, the presence of one or more variants of a combination in another, according to our criteria, cannot provide direct proof statistically or functionally that the combination in question is valid. A different variant combination, even with one shared variant, still implies different genetic and functional evidence. Therefore, every variant combination evidence is unique.

The information of shared variants is only taken into account for individual variant evidence and is subject to the discretion of the curators in the following cases: (1) as additional proof if this variant is present in controls and never found alone in patients, but always in combination with another variant, assuming a modifier role or (2) as additional proof that a variant is never found in controls and found only in patients, alone or with another variant, assuming a more prominent role, or (3) as additional proof if patients carrying variants in one particular gene (in combinations with another) have an earlier onset or more severe symptoms that patients not carrying this gene in a cohort study. This information provides some additional statistical proof of the relevance of a single variant, but can only be used in combination with the main part of the statistical proof of absence/presence in controls and based on the information of the other variants in the specific combination in question.

Similarly, for functional evidence, a reference to another article showing the functional effect of a single variant on gene function is considered relevant if the effect is linked to the phenotype.

## Post-curation data processing

### Annotating the data

After gathering and scoring all oligogenic variant combinations, the data went through several processing steps to formalise the gene and disease names, obtain the genomic coordinates of the variants and gather additional information for OLIDA. These processing steps only involved SNPs and indels. All scripts involved in this process are available upon request on https://github.com/oligogenic/olida_post_curation/.

The disease names were formalised using the Orphanet database (https://www.orpha.net/). Each oligogenic variant information was parsed to separate the variant and gene information. The genes were identified according to the gene nomenclature guidelines found on the database HGNC(8). The variants were then processed by the software Synvar (http://goldorak.hesge.ch/synvar/), in order to obtain the genomic coordinates, when possible. The variants that could not be mapped to the genome were manually annotated with the help of Varsome(9), a human genomic variation search engine, and dbSNP(10), a database of human genetic variation. Due to the inaccuracy of Synvar in certain cases or the incomplete nature of genomic variation databases, as well as the redundancy in gene names and symbols, we further annotated the variants with flags:

- automatically_attributed, if the variant was found and genomic coordinates were obtained using Synvar
- automatically_attributed_and_verified, if the variant was annotated by Synvar and was manually confirmed
- manually_attributed, if the variant could not be obtained using Synvar, but was found in a database
- manually_corrected, if the curators had to manually correct either the cDNA or protein change, due to incorrect or expired information from the relevant paper, and the resulting variant was also found in a database
- ambiguous_variant, if the variant found in the database needed a correction both in the cDNA and the protein change, thus making a decision impossible to take without further information from the authors of the relevant paper
- missing_from_databases, for the variants that we did not find in any database
- cnv, as CNVs are not in any of the aforementioned databases and do not typically have clearly defined genomic coordinates.

### Harmonization of the manual curation scores

During the manual curation process, we observed that the same gene combinations can have various gene combination evidence scores among different articles, as knowledge on gene relationships builds up over time or variable levels of information were described among papers. For example, most Bardet-Biedl syndrome genes are now known to be involved in the same protein complex(11,12), but this was not the case when the first articles suggesting oligogenic inheritance in this disease were published. To correct for this bias, for each gene pair, we defined a GENEmanual_harmonized score, corresponding to the highest corresponding gene combination manual score (GENEmanual) found among the curated papers and re-calculated the final manual curation confidence score for each combination involving this gene pair, based on the decision trees described in Supplementary Figures 1-2.

Furthermore, for an oligogenic combination described in multiple papers, we assigned the highest Manual score from each type of evidence found among the papers describing that combination and re-calculated its FINALmanual score, using the decision trees. This harmonisation can better depict the fact that different papers may focus their efforts in different aspects of proving the oligogenicity of a combination (e.g. one can focus on the genetic evidence of a genotyped pedigree and another on proving the synergy of the genes and variants with functional experiments).

### Knowledge scores

Even after harmonizing the functional evidence scores over the curated papers, a missing information bias still existed that could unfairly downgrade certain oligogenic combinations. Particularly in terms of the type of evidence we require for curation, we acknowledged that older papers did not have access to large biobanks and control cohorts (e.g. the 1000 Genomes Project(4)) and variant pathogenicity predictors, or the direct gene relationship information for the involved genes may not have been known or adequately described in the papers.

For this reason, we decided, for certain types of evidence, to create an additional type of score, the “knowledge score”, using information from public external databases that would compensate for any missing information in the curated papers. Specifically, we created knowledge scores for the statistical, functional gene combination and functional variant combination evidence. These scores were used, in combination with the manual curation scores, to define the final metascore of a combination, as it will be explained in the last section.

#### Genetic evidence - Statistical knowledge score (STATknowledge)

The statistical knowledge score compensates for any missing statistical information linked to the variants inside an oligogenic combination. This information exploits the main type of evidence that was used during the manual curation to assign scores. For this purpose, we checked the presence of each variant combination in the 1000 Genomes project(4) a database containing the genomic data of 2500 healthy individuals. We also checked their citations in ClinVar(13), to assess their link with the studied disease. It should be noted that the statistical knowledge score was only computed for SNPs and indels and in case all involved variants in a combination were annotated.

Based on this information, we defined the statistical knowledge score (STATknowledge) for a variant combination as:

| STATknowledge = | Weak (1), if the combination is not found as it is (i.e with the same zygosity status) in an individual of the 1000 Genomes Project(4) and the included variants are found in at least one other publication in ClinVar(13), excluding the publication where the oligogenic variant combination was extracted from.  Absent (0), if the combination is found as it is (i.e. with the same zygosity status) in an individual of the 1000 Genomes Project |
| --- | --- |

#### Functional evidence - Gene combination knowledge score (GENEknowledge)

The gene combination knowledge score compensates for any missing information on the gene relationship of the oligogenic combinations, by using relevant information from databases. This information exploits the main type of evidence that was used during the manual curation to assign scores: protein-protein interaction (PPI) information and pathway information, as the rest (co-localisation, expression in the same tissue) are not clear enough to provide an automated score on their own.

Protein-protein interaction information was obtained with the help of the comPPI database(14), a database of protein-protein interaction and colocalization. We used the confidence score of 0.8 of the interaction that is provided in the comPPI as a threshold for accepted interactions. The pathway information was retrieved from KEGG(15) and Reactome(16). We then manually screened each gene combination to determine if they have pathways or biological processes in common and verified whether these pathways are relevant for the studied phenotype.

Based on this information, we defined the gene combination knowledge score (GENEknowledge) as:

| GENEknowledge = | Moderate (2), if either one of the two conditions is met:   a) The gene combination forms a connected PPI network according to comPPI, and the comPPI score of each link is > 0.8 b) All members of the gene combination belong in the same Reactome or KEGG pathway/biological process, and this pathway/biological process is relevant for the studied phenotype   Weak (1), if all members of the gene combination belong in Reactome or KEGG pathways that are relevant for the studied phenotype, even if a link between them is not known.  Absent (0), otherwise |
| --- | --- |

#### Functional evidence - Variant combination knowledge score (VARknowledge)

The variant combination knowledge score compensates for any missing information on the pathogenic effect of the variants inside an oligogenic combination. This information exploits the main type of evidence that was used during the manual curation to assign scores. For this purpose, each variant was annotated with different pathogenicity predictors: SIFT(17), MutationTaster2(18), CADD(19) and Polyphen2(20). These tools were chosen for their easy access, wide usage in research papers, as well as the fact that they assess a different impact on the protein function. Due to the circularity of DIDA (and consequently OLIDA) being a training data set for VarCoPP, we did not use VarCoPP as a way to assess the pathogenicity of the variant combinations.

It should be noted that the statistical knowledge score was only computed for SNPs and indels and in case all involved variants in a combination were annotated.

Based on this information, we define the variant combination knowledge score (VARknowledge) as:

| VARknowledge = | Weak (1), if the VARmanual score for that combination is 0 and each variant is associated with a pathogenic effect from at least one aforementioned predictor. For CADD, the pathogenicity threshold is defined with the Phred value 15, and for Polyphen2 both “possibly damaging” and “probably damaging” values are accepted as indication of deleteriousness.  Absent (0), otherwise |
| --- | --- |

## Metascores

Finally, both manual and knowledge scores were combined, in order to create the confidence metascores for each type of evidence. For each oligogenic combination, statistical (STATmeta), gene pair (GENEmeta) and variant combination (VARmeta) evidence metascores were defined as the maximum score found between their corresponding manual and knowledge score. One exception occurred in this rule, where the STATknowledge score actually replaced the STATmanual score to define the STATmeta, in the case where the STATknowledge score is 0, due to the fact that the combination was found in the 1000 Genomes project(4). For the GENEmeta, the maximum of the GENEmanual_harmonized and the GENEknowledge was taken, since the GENEmanual_harmonized takes into account publication bias. The FAMmanual is kept as it is and is used for the calculation of the final metascore.

Once the individual evidence metascores were defined, the same process as in the manual curation was followed, in order to define the final confidence metascore (FINALmeta) for each combination. This time, instead of the manual curation scores, the metascores were used for each type of evidence. First, the gene and variant combination scores were combined to define the aggregated Functional Metascore (FUNmeta) by using the same decision tree as for the FUNmanual score, in Supplementary Figure 1. Then the FINALmeta score for each combination was defined, using the same decision tree as for the FINALmanual score, in Supplementary Figure 2. The FINALmeta score has exactly the same strength and meaning as the FINALmanual, as it is derived from the same decision trees but using more updated information:

| FINALmeta = | Strong (3), if we have a strong genetic evidence and proof of functional synergy among all involved genes and variants of the oligogenic combination, with a clear pathogenic synergistic effect on the studied phenotype.  Moderate (2), if we observe a good genetic segregation and functional synergy among all involved genes and variants of the oligogenic combination, showing an effect on the studied phenotype, but the described mechanism is still not clear or strong enough to provide proof of oligogenicity.  Weak (1), if the presented information on the oligogenic combination fulfills the basic requirements of a minimum presence of genetic and functional evidence to suggest that there is a pathogenic effect on the studied phenotype and that the variants do not occur by chance, but either there is no evidence of synergy or we are not certain that these are the only culprits for the studied phenotype.  Absent (0), for the rest of the oligogenic combinations that do not fulfill any of the aforementioned conditions. |
| --- | --- |

A summary of all scores used in the curation pipeline and post-curation can be found in Table 1.

# Supplementary Figures

Supplementary Figure 1. Decision tree that defines the FUNmanual and FUNmeta scores of a variant combination (where, in the second scenario, the VARmanual, GENEmanual and FUNmanual scores are replaced by VARmeta, GENEmeta and FUNmeta scores accordingly). The orange nodes represent an evidence score and the edges the value of this evidence score, while blue nodes represent decision nodes where the aggregated functional score is defined. The variant combination evidence is considered as the starting point (root) of the decision tree and, along with the information from the gene combination evidence, is used to define the final aggregated functional score. As the evidence at the variant level is very important in asserting whether a specific variant combination is functionally responsible for the observed phenotype, its absence (VARmanual = 0 ) can only be compensated by a very strong synergistic evidence at the gene level (GENEmanual = 3).

Supplementary Figure 2. Decision tree that defines the FINALmanual and FINAlmeta score of a variant combination (where, in the second scenario, all scores, apart from the FAMmanual, are replaced by their corresponding metascores). The orange nodes represent an evidence score and the edges the value of this evidence score, while blue nodes represent decision nodes where the final score is defined. The familial genetic score is considered as the starting point (root) of the decision tree and, along with the statistical score and functional score (whose value is defined by the decision tree in Supplementary Figure 1) is used to define the final score. Based on this decision tree, there is always a combination of genetic and functional evidence that is required to obtain a FINAL manual/meta score of 1, and the familial evidence (FAMmanual) is considered to be primarily important for this decision. A more lenient scenario is found in the case where the FAMmanual score is strong (=3), meaning that there is strong evidence of the pathogenicity of the studied variant combination based on the segregation of the involved variants in a large pedigree, as a variant combination can still be accepted with a final score of 1, if there is at least some minimum functional evidence at the gene level (GENEmeta/manual = 1).

Supplementary Figure 3. The database schema of OLIDA.

# Supplementary Bibliography

1.	Wilkinson, M. D., Dumontier, M., Aalbersberg, I. J. J., et al. (2016) The FAIR Guiding Principles for scientific data management and stewardship. *Sci Data*, 3, 160018.

2.	Schäffer, A. A. (2013) Digenic inheritance in medical genetics. *J. Med. Genet.*, 50, 641–652.

3.	Gazzo, A. M., Daneels, D., Cilia, E., et al. (2016) DIDA: A curated and annotated digenic diseases database. *Nucleic Acids Res.*, 44, D900–7.

4.	1000 Genomes Project Consortium, Auton, A., Brooks, L. D., et al. (2015) A global reference for human genetic variation. *Nature*, 526, 68–74.

5.	Karczewski, K. J., Francioli, L. C., Tiao, G., et al. (2020) The mutational constraint spectrum quantified from variation in 141,456 humans. *Nature*, 581, 434–443.

6.	Scott, E. M., Halees, A., Itan, Y., et al. (2016) Characterization of Greater Middle Eastern genetic variation for enhanced disease gene discovery. *Nat. Genet.*, 48, 1071–1076.

7.	Karczewski, K. J., Weisburd, B., Thomas, B., et al. The ExAC Browser: Displaying reference data information from over 60,000 exomes. Nucleic Acids Res., 45 (D1), D840-D845.

8.	Braschi, B., Denny, P., Gray, K., et al. (2019) Genenames.org: the HGNC and VGNC resources in 2019. *Nucleic Acids Res.*, 47, D786–D792.

9.	Kopanos, C., Tsiolkas, V., Kouris, A., et al. (2019) VarSome: the human genomic variant search engine. *Bioinformatics*, 35, 1978–1980.

10.	Sherry, S. T., Ward, M. H., Kholodov, M., et al. (2001) dbSNP: the NCBI database of genetic variation. *Nucleic Acids Res.*, 29, 308–311.

11.	Foggensteiner, L. and Beales, P. (2015) Bardet–Biedl syndrome and other ciliopathies. Bardet–Biedl syndrome and other ciliopathies. *Adv Exp Med Biol,* 1085*,* 171-174

12.	M’hamdi, O., Ouertani, I. and Chaabouni-Bouhamed, H. (2014) Update on the genetics of bardet-biedl syndrome. *Mol. Syndromol.*, 5, 51–56.

13.	Landrum, M. J., Lee, J. M., Benson, M., et al. (2018) ClinVar: improving access to variant interpretations and supporting evidence. *Nucleic Acids Res.*, 46, D1062–D1067.

14.	Veres, D. V., Gyurkó, D. M., Thaler, B., et al. (2015) ComPPI: a cellular compartment-specific database for protein-protein interaction network analysis. *Nucleic Acids Res.*, 43, D485–93.

15.	Kanehisa, M. and Goto, S. (2000) KEGG: Kyoto Encyclopedia of Genes and Genomes. *Nucleic Acids Res.*, 28, 27–30.

16.	Jassal, B., Matthews, L., Viteri, G., et al. (2020) The reactome pathway knowledgebase. *Nucleic Acids Res.*, 48, D498–D503.

17.	Kumar, P., Henikoff, S. and Ng, P. C. (2009) Predicting the effects of coding non-synonymous variants on protein function using the SIFT algorithm. *Nat. Protoc.*, 4, 1073–1081.

18.	Schwarz, J. M., Cooper, D. N., Schuelke, M., et al. (2014) MutationTaster2: mutation prediction for the deep-sequencing age. *Nat. Methods*, 11, 361–362.

19.	Rentzsch, P., Witten, D., Cooper, G. M., et al. (2019) CADD: predicting the deleteriousness of variants throughout the human genome. *Nucleic Acids Res.*, 47, D886–D894.

20.	Adzhubei, I. A., Schmidt, S., Peshkin, L., et al. (2010) A method and server for predicting damaging missense mutations. *Nat. Methods*, 7, 248–249.
